# Incidence, prevalence and mortality of patients with psoriasis: a U.K. population‐based cohort study

**DOI:** 10.1111/bjd.15021

**Published:** 2016-12-22

**Authors:** D.A. Springate, R. Parisi, E. Kontopantelis, D. Reeves, C.E.M. Griffiths, D.M. Ashcroft

**Affiliations:** ^1^ NIHR School for Primary Care Research (Centre for Primary Care)University of ManchesterStopford Building, Oxford RoadManchesterM13 9PTU.K; ^2^ Centre for BiostatisticsUniversity of ManchesterStopford Building, Oxford RoadManchesterM13 9PTU.K; ^3^ Centre for Pharmacoepidemiology and Drug SafetyManchester Pharmacy SchoolUniversity of ManchesterStopford Building, Oxford RoadManchesterM13 9PTU.K; ^4^ Centre for Health InformaticsInstitute of Population HealthUniversity of ManchesterStopford Building, Oxford RoadManchesterM13 9PTU.K; ^5^ Dermatology CentreSalford Royal NHS Foundation TrustUniversity of ManchesterManchester Academic Health Science CentreManchesterU.K

## Abstract

**Background:**

The burden of psoriasis across many world regions is high and there is a recognized need to better understand the epidemiology of this common skin disorder.

**Objectives:**

To examine changes in the prevalence and incidence of psoriasis, and mortality rates over a 15‐year period.

**Methods:**

Cohort study involving analysis of longitudinal electronic health records between 1999 and 2013 using the U.K. Clinical Practice Research Datalink (CPRD).

**Results:**

The prevalence of psoriasis increased steadily from 2·3% (2297 cases per 100 000) in 1999 to 2·8% (2815 per 100 000) in 2013, which does not appear to be attributable to changes in incidence rates. We observed peaks in age bands characteristic of early‐onset (type I) and late‐onset (type II) psoriasis, and changes in incidence and prevalence rates with increasing latitude in the U.K. All‐cause mortality rates for the general population and for patients with psoriasis have decreased over the last 15 years. However, the risk of all‐cause mortality for patients with psoriasis remains elevated compared with people without psoriasis (hazard ratio 1·21; 95% confidence interval 1·13–1·3) and we found no significant change in this relative excess mortality gap over time.

**Conclusions:**

We found an increasing population living longer with psoriasis in the U.K., which has important implications for healthcare service delivery and for resource allocation. Importantly, early mortality in patients with psoriasis remains elevated compared with the general population and we found no evidence of change in this premature mortality gap.

## Abstract 2

**What's already known about this topic?**

- The burden of psoriasis across many world regions is high and there is a need to better understand the epidemiology of this common skin disorder.

**What does this study add?**

- The prevalence of psoriasis in the U.K. is increasing and is higher than suggested from previous studies.
- A key driver to this increase relates to improvements in life expectancy rather than an increase in incidence of psoriasis over time.
- Despite improvements in life expectancy, patients with psoriasis remain more likely to die prematurely compared with those without psoriasis with no evidence of change in this premature mortality gap.

**Linked Comment:** Cust and Murrell. *Br J Dermatol* 2017; **176**:568–569.

Plain language summary available online

Psoriasis is a chronic inflammatory skin disease associated with high levels of psychosocial disability and impaired quality of life for prolonged periods.^1^, ^2^, ^3^ Our previous systematic review on the global epidemiology of psoriasis identified 53 published epidemiological studies reporting on the prevalence and/or incidence of psoriasis in the general population.^4^ In that review, we found estimates of the occurrence of psoriasis to vary markedly according to sex and geographic region. For example, reported adult psoriasis prevalence ranged from 1·3% in the U.K. [95% confidence interval (CI) 1·21–1·39]^5^ to 8·5% in Norway (95% CI 8·03–8·97).^6^ However, previous studies on the epidemiology of psoriasis have lacked consistency in case definition, thereby limiting the value of between‐country comparisons, and provide very limited data on temporal trends in the incidence and prevalence of this important skin disorder. Nonetheless, accurate and timely information on the epidemiology of psoriasis is needed in order to understand the impact of this disease and to ensure that adequate resources are provided nationally and regionally for people affected by psoriasis.^7^, ^8^

We also identified important knowledge gaps in understanding the natural history and burden of psoriasis.^4^ Specifically, very few studies have focused on the incidence of psoriasis and even fewer on trends in the incidence over time. To date, no studies have simultaneously compared longitudinal trends in incidence, prevalence and mortality in patients with psoriasis. This is critical in order to determine whether the prevalence of psoriasis is increasing over time, and if so, whether this is driven by increasing trends in incidence (more new cases of psoriasis) or whether patients are nowadays living much longer with psoriasis due to reductions in early mortality.

Several studies have reported on excess mortality in patients with psoriasis. For example, both cardiovascular mortality^9^ and all‐cause mortality^10^ have been reported to be elevated in patients with psoriasis. Over the last 30 years, though, overall survival in the general population in the U.K. has increased reflecting better overall population health.^11^ Given this, it is important to determine whether there have also been temporal changes in mortality in patients with psoriasis too, as this will impact on disease prevalence. As yet, it is unknown whether the mortality gap (the number of excess premature deaths) among patients with psoriasis is narrowing, widening or remaining unchanged over time.

Over 98% of the U.K. population are registered with a primary care general practitioner (GP)^12^ and under the National Health Service (NHS), visits to the GP are free of charge. U.K. primary care has been largely computerized since around 1998, when incentives for leaving paper‐based systems were provided.^13^ A number of large‐scale primary care databases have subsequently been developed, allowing researchers to answer important epidemiological questions using this routinely collected anonymized electronic health data. This study sets out to investigate the epidemiology of psoriasis in the U.K. using the Clinical Practice Research Datalink (CPRD), one of the largest U.K. primary care databases.^14^

Our aim was to determine trends in the incidence, prevalence and mortality of patients with psoriasis over 15 years in a large population‐based cohort study and examine how these epidemiological factors may have changed over time. In addition, we examined (i) whether there exists an association between latitude and incidence/prevalence; and (ii) whether or not the excess mortality in patients with psoriasis has changed over time.

## Methods

### Data source

We used the CPRD, a large primary care database that holds complete electronic patient records (including diagnoses, prescriptions, test results and hospital referrals) from participating family practices across the U.K. A hierarchical clinical coding system (Read) is used to record diagnosis data. In the database build we used (to July 2014), data were available for 685 practices and 15 436 637 patients.

### Psoriasis cohort

We extracted data from 1 January 1999 to 31 December 2013 and aggregated these into 15 separate years. Within each year, practice inclusion was determined by an internal CPRD data quality assessment algorithm. Practices that were rated as ‘up to standard for research purposes’ for the whole of a year were included for that year. Within each up‐to‐standard practice and year, all eligible patients had to be registered with the practice at the start of the year for follow‐up. Data on age, sex, neighbourhood deprivation, geographical region and removal from the database due to death or leaving the practice were available and complete for all patients. Neighbourhood deprivation was measured using the 2010 Index of Multiple Deprivation (IMD) for the practice postcode, categorized into quintiles; IMD is a composite score based on 38 indicators organized across seven different domains of deprivation (income, employment, health and disability, education, housing and services, living environment, and crime).^15^

Prevalent cases were those with at least one diagnostic psoriasis Read code in the database prior to the end of the year in question. Denominators for each year were all patients meeting the study eligibility criteria. Incident cases were patients with a first diagnosis Read code for psoriasis in the year in question. In calculating incidence rates, denominator counts were determined to be patients that were psoriasis‐free at the start of the year. Patients with prior codes for psoriasis or with codes for a history of psoriasis were excluded from both the numerator and denominator in determining incidence rates. For both incidence and prevalence, follow‐up time was calculated for each denominator case, so denominators could include patients who had died or transferred out of the practice mid‐year.

To investigate associations between psoriasis and mortality, for each person in our psoriasis incidence cohort we selected up to five control patients with no history of a diagnostic code for psoriasis, matched with regard to chronological age (5‐year bands), sex and practice. Each psoriasis case was assigned an index date based on the date of first diagnosis of psoriasis. All matched controls had to have had a consultation in the practice within ± 90 days of the case index date, with this set as the index date for the control, in order to ensure that patients with or without psoriasis were followed up by the same practice over similar time periods.

### Statistical analysis

All analyses were carried out using R version 3·1·2.^16^ Incidence and prevalence rates (95% CIs) were calculated with indirect adjustment for age and sex using the epiR package.^17^ All clinical code lists used in the study are available from www.clinicalcodes.org.^18^

We used Cox proportional hazards regressions to investigate mortality in the matched cohort study. The models included index year (as a continuous variable), sex, age at index date in 20‐year bands and practice level IMD quintiles. The analysis was clustered by practice. Cohort entry and exit times were collapsed into monthly time windows. A fully factorial model was initially fitted and the higher‐order interactions were removed in a stepwise fashion according to Akaike Information Criterion and individual coefficient *P*‐values. The proportional hazards assumption was assessed by scatterplots of the model coefficients over time and statistical testing based on weighted residuals.

As a sensitivity analysis, we repeated the analyses in a cohort restricted to include only practices that contributed data continuously for the whole of the period from 1 January 1999 to 31 December 2013.

Finally, we examined the relationship between latitude (measured at the central points of the 13 geographic regions in the CPRD consisting of the 10 regions in England plus Scotland, Wales and Northern Ireland) and incidence/prevalence rates, using mixed‐effects linear regressions, controlling for year and practice‐level deprivation. Practice was set to be a random effect in the analysis.

## Results

### Incidence and prevalence

**Figure 1:**
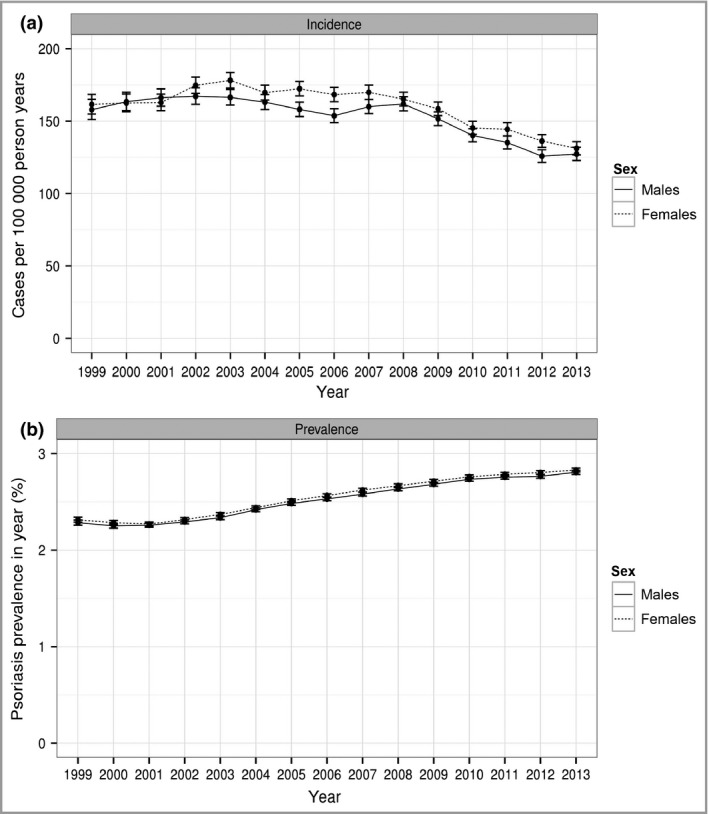
(a) Incidence and (b) prevalence (95% confidence intervals) of psoriasis from 1999 to 2013 for both males and females.

Yearly rates for incidence and prevalence of psoriasis, adjusted for age and sex differences between years are summarized in Table 1. Overall, adjusted psoriasis incidence declined from 159 cases per 100 000 person years (95% CI 155–164) in 1999 to 129 per 100 000 person years (95% CI 126–133) in 2013, although most of this decline occurred after 2008 (Fig. 1). Conversely, psoriasis prevalence rates increased from 2·3% (2297 cases per 100 000 person years) in 1999 to 2·8% (2815 per 100 000 person years) in 2013. Prevalence and incidence of psoriasis over time for both males and females, adjusted for year and sex, are shown in Figure 1. The patterns were similar in both males and females. Unadjusted incidence and prevalence rates based on raw counts are presented in Table S1 (see Supporting Information).

**Table 1:** Psoriasis prevalence and incidence per 100 000 person years; adjusted for age (95% confidence intervals)

Year	Total			Male			Female		
	Patients	Prevalence	Incidence	Patients	Prevalence	Incidence	Patients	Prevalence	Incidence
1999	2 842 495	2297 (2279–2315)	159 (155–164)	1 387 393	2284 (2258–2311)	158 (151–165)	1 455 102	2315 (2290–2341)	161 (155–168)
2000	3 510 428	2265 (2249–2282)	163 (158–167)	1 715 862	2251 (2228–2275)	163 (157–170)	1 794 566	2285 (2262–2308)	162 (156–169)
2001	4 053 693	2261 (2246–2276)	164 (160–168)	1 981 188	2258 (2236–2280)	166 (160–172)	2 072 505	2270 (2249–2292)	163 (157–168)
2002	4 524 286	2302 (2287–2316)	170 (166–174)	2 212 181	2293 (2272–2314)	166 (161–172)	2 312 105	2316 (2296–2336)	174 (169–180)
2003	4 910 335	2350 (2336–2364)	172 (168–176)	2 400 429	2336 (2316–2356)	166 (161–172)	2 509 906	2370 (2350–2390)	178 (173–183)
2004	5 219 699	2427 (2413–2441)	166 (163–170)	2 553 093	2419 (2399–2439)	163 (158–168)	2 666 606	2439 (2420–2459)	170 (165–175)
2005	5 467 744	2496 (2482–2509)	165 (162–169)	2 673 380	2484 (2464–2504)	158 (153–163)	2 794 364	2511 (2492–2530)	173 (168–178)
2006	5 590 290	2546 (2532–2559)	161 (158–165)	2 731 258	2530 (2510–2550)	154 (149–159)	2 859 032	2565 (2546–2584)	169 (164–174)
2007	5 657 122	2598 (2585–2612)	165 (162–168)	2 763 463	2580 (2560–2600)	160 (155–165)	2 893 659	2622 (2602–2641)	170 (165–175)
2008	5 784 107	2648 (2635–2662)	163 (160–167)	2 823 447	2634 (2614–2653)	162 (157–167)	2 960 660	2668 (2648–2687)	165 (160–170)
2009	5 808 529	2695 (2682–2709)	155 (152–158)	2 832 154	2682 (2662–2702)	152 (147–156)	2 976 375	2713 (2694–2732)	159 (154–163)
2010	5 724 132	2744 (2730–2758)	143 (140–146)	2 786 062	2734 (2714–2754)	140 (136–145)	2 938 070	2760 (2740–2779)	145 (141–150)
2011	5 694 515	2767 (2753–2781)	140 (137–143)	2 766 330	2755 (2735–2776)	135 (131–140)	2 928 185	2785 (2766–2805)	144 (140–149)
2012	5 648 960	2781 (2767–2795)	131 (128–134)	2 738 101	2765 (2745–2785)	126 (122–130)	2 910 859	2803 (2783–2823)	136 (132–141)
2013	5 226 764	2815 (2800–2830)	129 (126–133)	2 525 879	2806 (2785–2827)	127 (123–132)	2 700 885	2830 (2810–2851)	131 (127–136)

**Figure 2:**
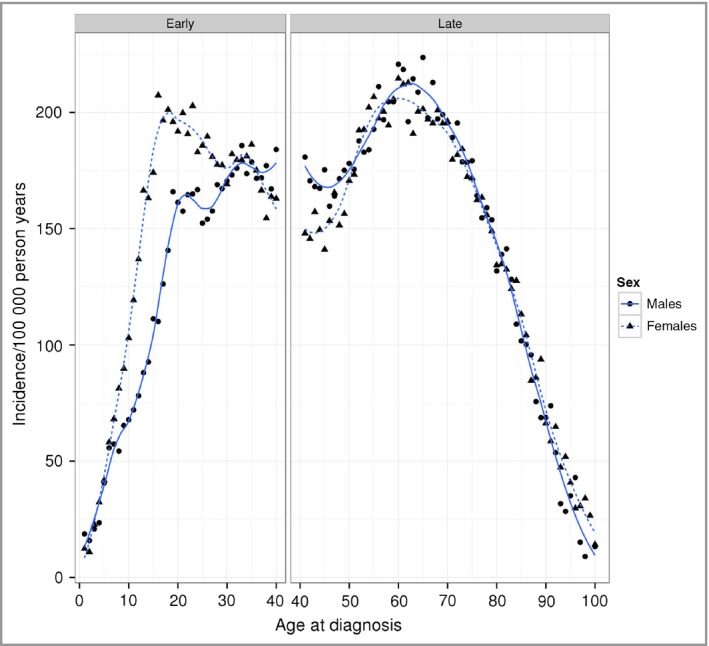
Incidence of psoriasis by age at diagnosis for males and females, with Loess smoothers (males, solid line; females, dashed line). The graph has been split into panels for early‐ and late‐onset psoriasis.

Psoriasis incidence plotted against age showed a strongly bimodal pattern (Fig. 2). ‘Late‐onset’ psoriasis (categorized as being diagnosed > 40 years of age) shows little difference in distribution between males and females. However, ‘early‐onset’ psoriasis showed clear differences with females being more likely to be diagnosed with psoriasis at an earlier age.

**Figure 3:**
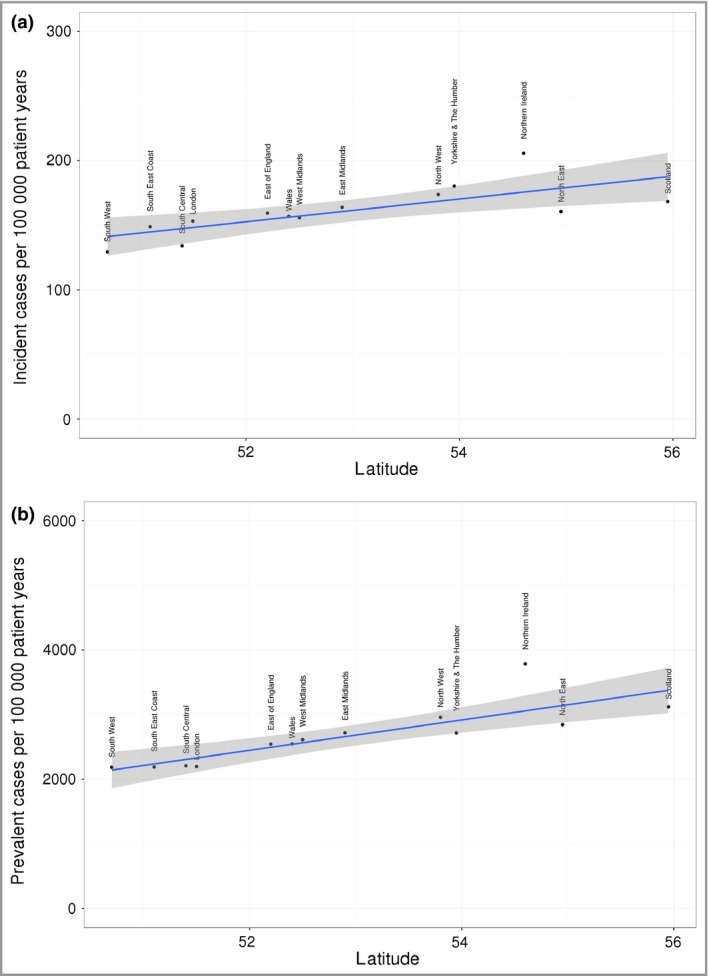
Relationship between latitude and (a) incidence and (b) prevalence of psoriasis.

Both incidence and prevalence of psoriasis showed a positive relationship with latitude (Fig. 3). Controlling for year, deprivation and practice, incidence increased by 6·5 cases per 100 000 for every degree increase in latitude (95% CI 4–9·1 cases per 100 000) and prevalence increased by 201 cases per 100 000 for every degree in latitude (95% CI 163·5–238·6 cases per 100 000). In South West England, mean psoriasis incidence was 144 (95% CI 138–150 cases per 100 000) and mean prevalence was 2323 (95% CI 2266–2380 cases per 100 000). In Scotland, mean psoriasis incidence was 174 (95% CI 167–181 cases per 100 000) and mean prevalence was 3060 (95% CI 2991–3128 cases per 100 000).

Similar rates and trends in incidence and prevalence were observed when restricting the cohort to include patients only from practices contributing data for the whole period between 1999 and 2013 as a planned sensitivity analysis (Fig. S1, see Supporting Information).

### Mortality

The characteristics of the matched psoriasis cases and controls are summarized in Table 2. Median follow‐up time was similar for psoriasis cases and controls (mean follow‐up time = 64 months).

**Table 2:** Summary of characteristics of patients included in the mortality analysis (matched on practice, age, sex)

	Psoriasis cases	Controls
*n*	104 441	508 457
Male patients (% of *n*)	49 787 (47·7%)	241 918 (47·6%)
Deaths (% of *n*)	6555 (6·3%)	29 395 (5·8%)
Follow‐up (person years)	6 693 444	32 732 726
Mean months follow‐up (SD)	64·1 (45)	64·4 (45·2)
Age (% of *n*), years		
0–19	12 633 (12·1%)	66 048 (13%)
20–39	30 946 (29·6%)	147 051 (28·9%)
40–59	32 829 (31·4%)	160 319 (31·5%)
60–79	23 810 (22·8%)	115 165 (22·6%)
≥ 80	4223 (4%)	19 874 (3·9%)
Index year (% of *n*)		
1999	3959 (3·8%)	20 202 (4%)
2000	4951 (4·7%)	24 275 (4·8%)
2001	5806 (5·6%)	28 564 (5·6%)
2002	6740 (6·5%)	33 226 (6·5%)
2003	7460 (7·1%)	36 329 (7·1%)
2004	7723 (7·4%)	37 543 (7·4%)
2005	7995 (7·7%)	39 012 (7·7%)
2006	7985 (7·6%)	38 992 (7·7%)
2007	8274 (7·9%)	40 320 (7·9%)
2008	8485 (8·1%)	41 008 (8·1%)
2009	8058 (7·7%)	39 116 (7·7%)
2010	7336 (7%)	35 630 (7%)
2011	7182 (6·9%)	34 554 (6·8%)
2012	6703 (6·4%)	32 367 (6·4%)
2013	5784 (5·5%)	27 319 (5·4%)
Practice‐level deprivation quintile (% of *n*)		
1 (least deprived)	18 908 (18·1%)	92 132 (18·1%)
2	20 231 (19·4%)	98 468 (19·4%)
3	21 436 (20·5%)	104 330 (20·5%)
4	21 539 (20·6%)	104 793 (20·6%)
5 (most deprived)	22 327 (21·4%)	108 734 (21·4%)

**Figure 4:**
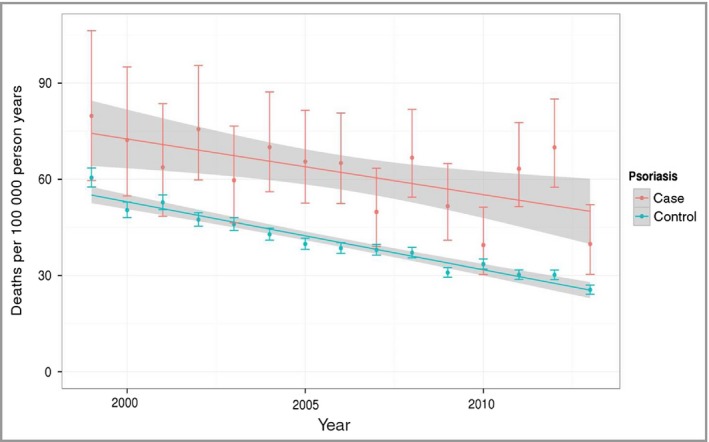
Temporal trends in mortality in patients with psoriasis (case) and without psoriasis (control).

The results of the mortality analysis are shown in Table 3. We observed a significant interaction between psoriasis and age at index date. Patients in the reference age category (40–59 years) with psoriasis had a 20% higher mortality rate than their matched controls [hazard ratio (HR) 1·2, 95% CI 1·12–1·28]. In the youngest group (0–19 years), increased mortality associated with psoriasis appeared even higher, although small numbers of patients in this group resulted in wider confidence intervals (HR 1·53, 95% CI 1·02–2·3). Mortality among patients diagnosed with psoriasis at an older age did not appear to be increased (age 60–79 years: HR 1·08, 95% CI 0·99–1·16; age 80+ years: HR 0·99, 95% CI 0·91–1·08). There was an overall reduction in mortality over time for all patients (HR 0·92 per year, 95% CI 0·91–0·92), with no evidence that this differed between those with and without psoriasis (i.e. no significant interaction). Figure 4 shows reductions in mortality rates for patients with and without psoriasis over the 15‐year study period. Results were similar when we limited the cohort to include only those practices that contributed data for the entire period of the study (Table S2, see Supporting Information). In this sensitivity analysis, the HR for psoriasis in the reference group was 1·25 (95% CI 1·13–1·37).

**Table 3:** Results of the Cox regression analysis examining the risk of mortality in patients with psoriasis. ‘Age’ represents age at index date; age 40–59 is the reference category)

Variable	HR (95% CI)	Coefficient (se)	z	*P*–value
Index year	0·92 (0·91–0·92)	–0·086 (0·002)	–40·218	**< 0·0001**
Women	0·7 (0·66–0·74)	–0·36 (0·029)	–12·439	**< 0·0001**
Age 0–19	0·07 (0·05–0·09)	–2·667 (0·122)	–21·926	**< 0·0001**
Age 20–39	0·22 (0·2–0·24)	–1·531 (0·05)	–30·598	**< 0·0001**
Age 60–79	5·6 (5·34–5·87)	1·722 (0·024)	70·981	**< 0·0001**
Age ≥ 80	25·2 (23·71–26·83)	3·228 (0·032)	102·398	**< 0·0001**
IMD 2^a^	1·07 (1·01–1·14)	0·072 (0·031)	2·302	**0·0213**
IMD 3^a^	1·12 (1·06–1·18)	0·113 (0·028)	3·997	**0·0001**
IMD 4^a^	1·22 (1·14–1·29)	0·195 (0·031)	6·218	**< 0·0001**
IMD 5^a^	1·38 (1·3–1·47)	0·324 (0·032)	10·243	**< 0·0001**
Psoriasis	1·21 (1·13–1·3)	0·19 (0·036)	5·374	**< 0·0001**
Female : Age 0–19^b^	0·75 (0·53–1·06)	–0·288 (0·178)	–1·623	0·1045
Female : Age 20–39^b^	0·85 (0·74–0·98)	–0·164 (0·073)	–2·234	**0·0255**
Female : Age 60–79^b^	1·01 (0·95–1·08)	0·013 (0·033)	0·382	0·7022
Female : Age ≥ 80^b^	1·11 (1·03–1·21)	0·108 (0·04)	2·693	**0·0071**
Age 0–19 : psoriasis^b^	1·27 (0·85–1·89)	0·236 (0·204)	1·156	0·2479
Age 20–39 : psoriasis^b^	0·88 (0·73–1·06)	–0·129 (0·094)	–1·373	0·1698
Age 60–79 : psoriasis^b^	0·89 (0·83–0·96)	–0·115 (0·04)	–2·896	**0·0038**
Age ≥ 80 : psoriasis^b^	0·82 (0·75–0·9)	–0·199 (0·044)	–4·54	**< 0·0001**
Index year : psoriasis^b^	1·01 (1–1·01)	0·005 (0·004)	1·256	0·209

CI, confidence interval; HR, hazard ratio; IMD, Index of Multiple Deprivation, quintile. *P*‐values significant at the 0·05 level are marked in bold. ^a^Quintile 1 (least deprived) is the reference category. ^b^Interactions between variables are indicated by a colon.

## Discussion

We found that over a 15‐year period (between 1999 and 2013), psoriasis prevalence steadily increased and our latest estimates suggest that psoriasis now affects over 2·8% of the general population in the U.K. At the same time, mortality in patients with psoriasis and to a lesser extent incident cases of psoriasis has decreased. All‐cause mortality rates for patients with psoriasis remain elevated compared with patients without psoriasis and we found no significant change in this relative premature mortality gap over time. These findings show that there is an increasing population living longer with psoriasis, which has important implications for healthcare service delivery. Within the U.K., we observed positive associations between incidence and prevalence of psoriasis and latitude. We also found a clear bimodal pattern in incidence of psoriasis when examined against patient age, supporting the notion of ‘type I’ (early‐onset) and ‘type II’ (late‐onset) variants of the condition.

An increasing trend in psoriasis prevalence has been observed in several settings before, for example in the U.S.A., Norway and Spain.^19^, ^20^, ^21^, ^22^, ^23^ This observed increase in prevalence of psoriasis has been speculated to be related to a better awareness of the disease among physicians and in the general population (possibly due to the advent of biologic therapies) rather than a real increase in the prevalence of the disease.^23^ In this study, we were able to consider this steady increase in psoriasis in the context of a decreasing risk of mortality. As fewer patients die for every incident case over time, we found that the prevalence pool of patients with psoriasis is steadily increasing.

Studies of the incidence of psoriasis longitudinally are scarce. Results from two earlier cross‐sectional studies in the U.S.A. suggested an increasing trend of incident cases of psoriasis over a 30‐year period both in children^24^ and in adults.^25^ The reasons behind the suggested increasing trend were unknown, but included a variety of potential explanations, among which were a true change in incidence of psoriasis, a change in the diagnosis patterns over time^25^ or an increase in risk factors for psoriasis such as obesity.^24^ Our findings relating to age of onset of psoriasis show a clear separation between incidence of early‐ and late‐onset psoriasis (‘type I’ and ‘type II’), corresponding to whether the diagnosis is made at ≤ 40 or > 40 years of age.^26^ In early‐onset psoriasis, females have a higher incidence of psoriasis and the peak of early‐onset psoriasis occurs in their late teens and early twenties. This pattern reflects previously reported more rapid increase in psoriasis prevalence in women.^27^ The corresponding peak of early‐onset psoriasis in men appeared later – in their thirties. This difference was not observed in late‐onset psoriasis in which the pattern of incidence by age did not appear to differ between males and females.

However, latitude appeared to have a significant effect on psoriasis, with around 6·5 new psoriasis cases per 100 000 for every degree increase in latitude in the U.K. Our earlier systematic review of the global epidemiology of psoriasis noted that the prevalence of psoriasis varied between geographic regions, with psoriasis appearing more commonly in countries more distant from the equator.^4^ Likely mechanisms for these findings include the degree of solar irradiance and the metabolism of vitamin D. Vitamin D analogues are well established as an effective treatment for mild‐to‐moderate psoriasis when applied topically and phototherapy has been used widely to treat psoriasis that cannot be controlled with topical treatment alone. Further studies of the effects of solar irradiance and latitude on the incidence and prevalence of psoriasis in other settings are needed to confirm this relationship with disease epidemiology.

To our knowledge, we present the first study to examine simultaneously trends in incidence, prevalence and mortality of patients with psoriasis over a prolonged period in a large representative sample of the general population. In considering these epidemiological measures concurrently, we found that the increasing survival of patients with psoriasis is contributing to the increased prevalence of the condition in the U.K. Although the size and validity of the database used allowed us to investigate these trends effectively, some important limitations remain that should be considered alongside our findings. Firstly, psoriasis cases were identified from general practice electronic health records using relevant diagnostic code lists and so may not necessarily have been verified by dermatologists. However, in the U.K., primary care is fully computerized and appropriate coding in clinical computer systems is considered standard practice.^28^ Previous studies have shown that primary care electronic health records in the U.K. are a valid data resource for studying psoriasis.^29^, ^30^ A limitation, nonetheless, is that our study includes only those patients who present in general practice and thereby receive a physician diagnosis of psoriasis, but this would also be true in other patient populations.

For the first time, we provide a comprehensive longitudinal picture of the prevalence and incidence of psoriasis, and mortality rates over a 15‐year period. We present a rise in the prevalence of diagnosed psoriasis in the U.K., between 1999 and 2013, which does not appear to be attributable to a corresponding increase in incidence. We found that there is an increasing population living longer with psoriasis in the U.K., which has resulted in an increase in the prevalence and this has important implications for healthcare service delivery and for resource allocation. We observed peaks in age bands characteristic of early‐ and late‐onset psoriasis, and changes in incidence and prevalence rates with increasing latitude. All‐cause mortality rates for patients with psoriasis have decreased over the last 15 years, but remain elevated compared with patients without psoriasis. Importantly, we found no significant change in this premature mortality gap over time.

## Supporting information


**Table S1.** Unadjusted incidence and prevalence numerators, denominators and rates. Values are shown for males and females split by year.
**Table S2.** Results of the Cox regression analysis examining the risk of mortality in patients with psoriasis in the cohort reduced to those practices contributing data continuously between 1999 and 2013.
**Fig S1.** Incidence and prevalence (95% confidence intervals) of psoriasis in continuously contributing Clinical Practice Research Datalink practices from 1999 to 2013 for both men and women.
