# Supplementary material for: Incidence, prevalence and mortality of patients with psoriasis: a U.K. population‐based cohort study
Source: Br J Dermatol. 2016 Dec 22;176(3):650–8. doi: 10.1111/bjd.15021 (PMC5363241; doi:10.1111/bjd.15021)
Supplement: Supplementary file 1 — Table S1. Unadjusted incidence and prevalence numerators, denominators and rates. Values are shown for males and females split by year. Table S2. Results of the Cox regression analysis examining the risk of mortality in patients with psoriasis in the cohort reduced to those practices contributing data continuously between 1999 and 2013. Fig S1. Incidence and prevalence (95% confidence intervals) of psoriasis in continuously contributing Clinical Practice Research Datalink practices from 1999 to 2013 for both men and women. [file BJD-176-650-s001.docx]

# Supplementary material

|  | Incidence | | | | | | Prevalence | | | | | |
| --- | --- | --- | --- | --- | --- | --- | --- | --- | --- | --- | --- | --- |
|  | Men | | | Women | | | Men | | | Women | | |
| year | numerator | denominator | rate | numerator | denominator | rate | numerator | denominator | rate | numerator | denominator | rate |
| 1999 | 2069 | 1312205 | 157.7 | 2210 | 1372835 | 161.0 | 29539 | 1307670 | 2258.903 | 31875 | 1366975 | 2331.8 |
| 2000 | 2659 | 1626364 | 163.5 | 2739 | 1696456 | 161.5 | 35996 | 1616879 | 2226.264 | 38706 | 1684236 | 2298.1 |
| 2001 | 3111 | 1874703 | 165.9 | 3175 | 1957438 | 162.2 | 41744 | 1869503 | 2232.893 | 44548 | 1949212 | 2285.4 |
| 2002 | 3467 | 2090948 | 165.8 | 3792 | 2179800 | 174.0 | 47248 | 2083339 | 2267.898 | 50597 | 2169282 | 2332.4 |
| 2003 | 3774 | 2270780 | 166.2 | 4203 | 2367920 | 177.5 | 52469 | 2266823 | 2314.649 | 56403 | 2360673 | 2389.3 |
| 2004 | 3941 | 2420676 | 162.8 | 4268 | 2522699 | 169.2 | 57195 | 2352899 | 2430.831 | 61318 | 2449412 | 2503.4 |
| 2005 | 3994 | 2529732 | 157.9 | 4528 | 2639822 | 171.5 | 61746 | 2470452 | 2499.381 | 66444 | 2575935 | 2579.4 |
| 2006 | 3970 | 2579328 | 153.9 | 4529 | 2694615 | 168.1 | 64306 | 2516255 | 2555.623 | 69366 | 2626419 | 2641.1 |
| 2007 | 4184 | 2609211 | 160.4 | 4623 | 2726537 | 169.6 | 66557 | 2546874 | 2613.282 | 71896 | 2658343 | 2704.5 |
| 2008 | 4345 | 2675581 | 162.4 | 4619 | 2802579 | 164.8 | 69941 | 2608647 | 2681.122 | 75369 | 2731497 | 2759.3 |
| 2009 | 4076 | 2679532 | 152.1 | 4442 | 2812588 | 157.9 | 71786 | 2620810 | 2739.077 | 77307 | 2750747 | 2810.4 |
| 2010 | 3706 | 2631112 | 140.9 | 4009 | 2772498 | 144.6 | 71853 | 2567517 | 2798.54 | 77524 | 2708345 | 2862.4 |
| 2011 | 3538 | 2609357 | 135.6 | 3961 | 2762919 | 143.4 | 71999 | 2549368 | 2824.19 | 78099 | 2700771 | 2891.7 |
| 2012 | 3278 | 2592704 | 126.4 | 3714 | 2754030 | 134.9 | 71807 | 2527435 | 2841.102 | 78215 | 2684687 | 2913.4 |
| 2013 | 3038 | 2379369 | 127.7 | 3312 | 2545459 | 130.1 | 67295 | 2325019 | 2894.385 | 73286 | 2486000 | 2947.9 |

**Table S1 Unadjusted incidence and prevalence numerators, denominators and rates. Values are shown for men and women split by year.**

|  | HR (95%CI) | coefficient (se) | z | P-value |
| --- | --- | --- | --- | --- |
| Index year | 0.92 (0.91-0.92) | -0.085 (0.003) | -30.373 | **<0.0001** |
| Women | 0.68 (0.63-0.74) | -0.389 (0.041) | -9.404 | **<0.0001** |
| Age 0-19 | 0.06 (0.04-0.08) | -2.84 (0.184) | -15.454 | **<0.0001** |
| Age 20-39 | 0.21 (0.19-0.24) | -1.558 (0.064) | -24.471 | **<0.0001** |
| Age 60-79 | 5.6 (5.24-5.99) | 1.723 (0.034) | 50.086 | **<0.0001** |
| Age >=80 | 24.04 (21.94-26.35) | 3.18 (0.047) | 68.064 | **<0.0001** |
| IMD 2 | 1.06 (0.98-1.14) | 0.056 (0.037) | 1.518 | 0.12908 |
| IMD 3 | 1.11 (1.03-1.18) | 0.101 (0.034) | 2.952 | **0.00316** |
| IMD 4 | 1.21 (1.1-1.32) | 0.19 (0.046) | 4.083 | **<0.0001** |
| IMD 5 | 1.38 (1.28-1.5) | 0.326 (0.041) | 7.914 | **<0.0001** |
| Psoriasis | 1.25 (1.13-1.37) | 0.22 (0.048) | 4.616 | **<0.0001** |
| Women:Age 0-19 | 0.61 (0.33-1.12) | -0.497 (0.313) | -1.587 | 0.11253 |
| Women:Age 20-39 | 0.96 (0.79-1.15) | -0.045 (0.095) | -0.477 | 0.63314 |
| Women:Age 60-79 | 1.03 (0.94-1.13) | 0.03 (0.047) | 0.654 | 0.51315 |
| Women:Age >=80 | 1.19 (1.07-1.33) | 0.178 (0.055) | 3.261 | **0.00111** |
| Age 0-19:psoriasis | 1.34 (0.74-2.42) | 0.29 (0.303) | 0.958 | 0.3379 |
| Age 20-39:psoriasis | 0.87 (0.67-1.13) | -0.139 (0.134) | -1.033 | 0.30153 |
| Age 60-79:psoriasis | 0.85 (0.77-0.94) | -0.163 (0.053) | -3.082 | **0.00206** |
| Age >=80:psoriasis | 0.78 (0.69-0.88) | -0.25 (0.06) | -4.176 | **<0.0001** |

**Table S2 Results of the Cox regression analysis examining the risk of mortality in patients with psoriasis in cohort reduced to those practices contributing data continuously between 1999 to 2013.**


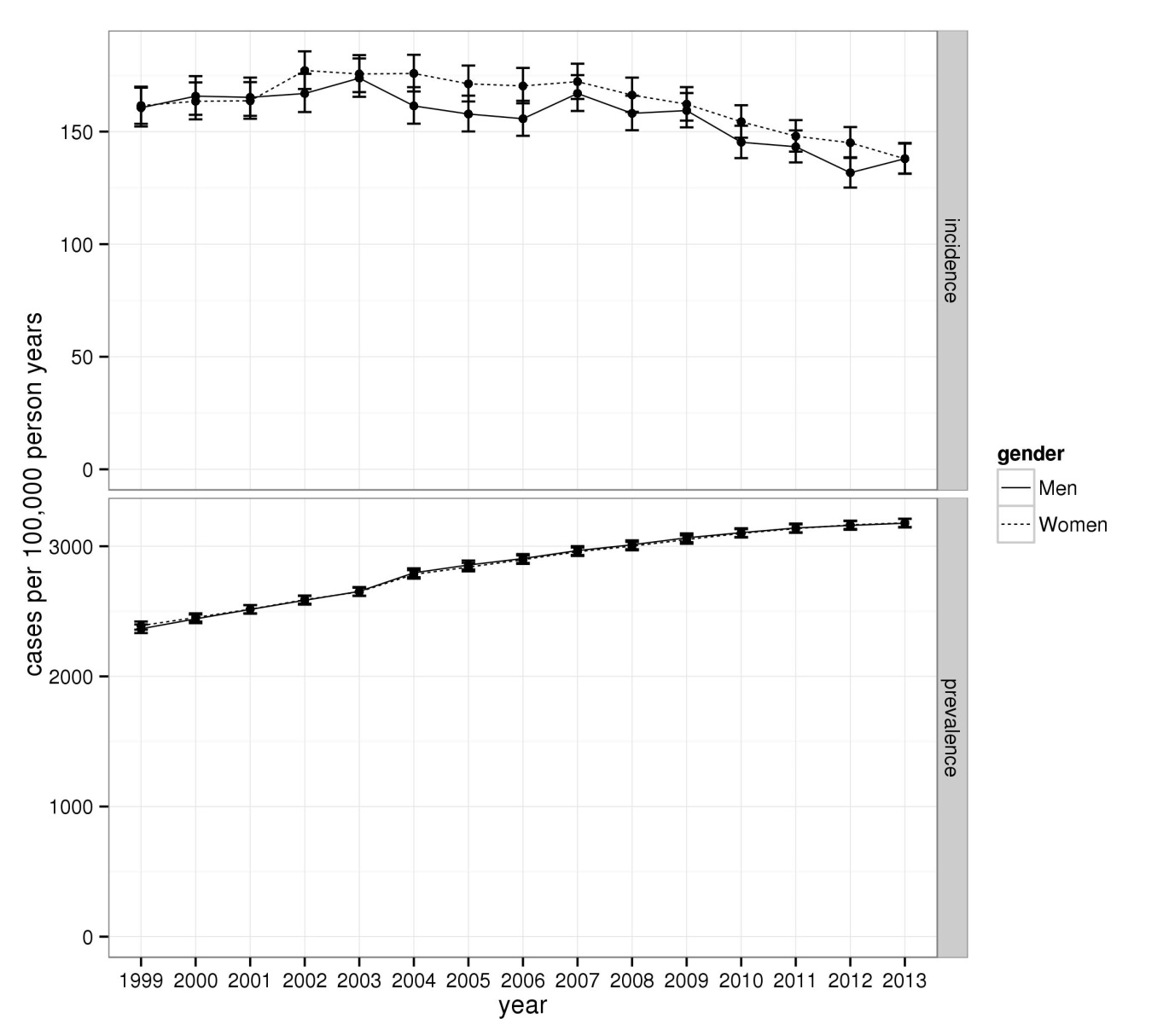
**Figure S1 . Incidence and prevalence (95% Confidence intervals) of Psoriasis in continuously contributing CPRD practices from 1999 to 2013 for both men and women.**
